# Establishment of a Poliovirus Containment Program and Containment Certification Process for Poliovirus-Essential Facilities, United States 2017–2022

**DOI:** 10.3390/pathogens13020116

**Published:** 2024-01-27

**Authors:** Christy Ottendorfer, Bryan Shelby, Cecelia A. Sanders, Anna Llewellyn, Christy Myrick, Christye Brown, Suganthi Suppiah, Kortney Gustin, Lia Haynes Smith

**Affiliations:** 1 Centers for Disease Control and Prevention, Office of Readiness and Response, U.S. National Authority for Containment of Poliovirus, Atlanta, GA 30329, USA; uyk0@cdc.gov (C.O.); zfg0@cdc.gov (C.A.S.);; 2 Centers for Disease Control and Prevention, National Center for Emerging and Zoonotic Infectious Diseases, Atlanta, GA 30329, USA; gif7@cdc.gov; 3 Centers for Disease Control and Prevention, Office of Laboratory Science and Safety, Atlanta, GA 30329, USA; 4 Morehouse School of Medicine, Atlanta, GA 30310, USA; cybrown@msm.edu

**Keywords:** polio, GAPIII, poliovirus containment, NAC, poliovirus-essential facility, PEF

## Abstract

Upon declaration of poliovirus (PV) type 2 eradication in 2015, the World Health Organization (WHO) published PV containment requirements in the Global Action Plan III (GAPIII) for mitigating the risk of a facility-associated release post eradication. In 2018, the 71st World Health Assembly resolution urged member states retaining PV to appoint a National Authority for Containment (NAC), reduce the number of PV facilities, and submit applications for containment certification. The United States (US) NAC was established in 2018 for containment oversight, and two paths to WHO GAPIII containment certification were developed. Facilities retaining PV were identified through national poliovirus containment surveys. The US NAC conducted 27 site visits at 18 facilities (20 laboratories: A/BSL-2 (65%), A/BSL-3 (20%), and storage-only (15%)) to verify the implementation of US NAC’s preliminary containment measures. The NAC identified areas for improvement in seven categories: primary containment, decontamination, hand hygiene, security, emergency response, training, and immunization practices. Sixteen facility applications were endorsed to pursue poliovirus-essential facility (PEF) certification, whereas four facilities opted to withdraw during the containment certification process. The US made noteworthy progress in PV containment to enhance biosafety and biosecurity practices at US PV facilities to safeguard the polio eradication effort.

## 1. Introduction

Two of the three wild poliovirus (WPV) serotypes, type 2 and type 3, were declared eradicated in 2015 and 2019, respectively [1]. Wild poliovirus type 1 remains endemic in only two countries (Afghanistan and Pakistan) [1]. In the United States (US), the last wild poliovirus outbreak occurred in 1979 [2]. Wild polioviruses were eliminated in the US due to successful childhood immunization programs with the live-attenuated oral poliovirus vaccine (OPV) that was later replaced with an inactivated injectable intramuscular formulation in 2000 [2,3]. Since the Global Polio Eradication Initiative (GPEI) was founded in 1988, the global incidence of wild poliovirus has declined from 350,000 cases a year in 176 countries to 22 cases in 2 countries in 2022, with an additional 8 cases in Mozambique linked to importation of virus circulating in Pakistan [1]. Globally, continued use of OPV contributed to outbreaks of neurovirulent circulating vaccine-derived poliovirus (cVDPV) in areas with low population immunity [1]. Since most cVDPV outbreaks were caused by OPV/Sabin strain type 2 (OPV2) [4], World Health Organization (WHO) member states synchronized a global switch in April 2016 from trivalent OPV (tOPV, contains Sabin strain types 1, 2, 3) to bivalent OPV (bOPV, contains Sabin strain types 1 and 3) for routine and supplementary immunization programs [5]. The global cessation of routine OPV2 use triggered stringent containment requirements for all poliovirus type 2 (PV2) materials to minimize the risk of reintroduction of PV2 from facilities back into communities [6,7,8,9].

The *WHO Global Action Plan to minimize poliovirus-facility-associated risk after type-specific eradication of wild polioviruses and sequential cessation of oral polio vaccine use*, 3rd ed. (GAPIII), outlines facility containment requirements to mitigate the risks of a facility-associated release of poliovirus [10], and in 2022, WHO released the *Global Action Plan for Poliovirus Containment*, 4th ed. (GAPIV) [11], to address public comments received on GAPIII with full implementation anticipated by 2026. The World Health Assembly (WHA) resolution 71.16 in May 2018 urged countries retaining polioviruses to (1) reduce the number of facilities, (2) appoint a National Authority for Containment (NAC) for oversight of containment activities, and (3) have facilities selected to retain polioviruses to submit applications for participation in the containment certification program no later than December 2019 [12]. Countries are working to implement GAPIII/GAPIV containment guidelines including appointing NACs and conducting national surveys to identify facilities retaining poliovirus infectious materials (e.g., seed stocks and virus isolates) and potentially infectious materials (PIMs [13], e.g., stool or upper respiratory specimens not known to contain poliovirus that were collected in a time and place when poliovirus was circulating or OPV was used) [6]. Further, countries must identify and approve a limited number of facilities as designated poliovirus-essential facilities (PEFs) permitted to retain polioviruses in accordance with GAPIII/GAPIV [10,11] and applicable national laws, regulations, or standards. These facilities must be certified against GAPIII/GAPIV requirements via an auditing process described in the WHO GAPIII Containment Certification Scheme (CCS) [14]. The WHO and GPEI’s Global Commission for the Certification of Poliomyelitis Eradication (GCC) provides oversight and uses an independent committee, the GCC Containment Working Group (GCC-CWG), to make recommendations to the GCC for endorsement of PEF containment certificates. A GCC-endorsed and NAC-countersigned application provides assurance to the global community that the facility’s implementation of GAPIII/GAPIV is appropriate and consistent worldwide [14].

While US poliovirus survey activities began in 2002 with the first national survey [15], in January 2017, the Poliovirus Containment Activity was established within the U.S. Centers for Disease Control and Prevention (CDC) to perform the global poliovirus containment functions in the United States. In January 2018, the US Department of Health and Human Services Acting Assistant Secretary of Health approved the designation of the Poliovirus Containment Activity as the US NAC. The US NAC operates with eight full-time government employees responsible for the national poliovirus inventory (surveys) and oversight of GAPIII containment certification. The US NAC’s director also has oversight responsibilities as the US National Poliovirus Containment Coordinator, who oversees the national survey and inventory. We report the establishment of a national poliovirus containment program, describe a voluntary containment certification process for poliovirus-essential facilities, and share preliminary results of poliovirus laboratory containment implementation in the United States.

## 2. Materials and Methods

### 2.1. US Poliovirus Containment Program

A national program was established within the CDC Office of Readiness and Response to provide oversight of poliovirus containment in the United States. US NAC created a website, guidance, and a containment certification process for facilities retaining eradicated poliovirus materials. PV2 infectious materials (i.e., wild, vaccine-derived poliovirus (VDPV), OPV/Sabin viruses) and, since 2019, wild and vaccine-derived poliovirus type 3 materials (WPV3/VDPV3) are subject to WHO’s GAPIII containment requirements and should be held only in PEFs [16]. Poliovirus-essential facility containment certification is overseen by the US NAC audit team. The audit team is composed of four auditors with expertise in microbiology and biosafety, and prior experience performing regulatory inspections of US select agent laboratories. Auditors also completed the WHO GAPIII auditor training course and maintain ISO45001:2018 [17] and ISO19011:2018 [18] certifications. Auditors disclose any potential conflicts of interest to the NAC Director consistent with the certification scheme [14].

### 2.2. Facility Identification and Outreach

US facilities retaining PV materials were identified through three national surveys conducted by CDC between 2002 and 2022, which were used to determine the national inventory of PV materials [15,19,20]. US NAC used survey results to identify and contact facilities reporting PV materials and perform outreach with webinars and site visits to provide training on WHO GAPIII containment certification. Facilities not entering the WHO Containment Certification Scheme submitted documentation to the US NAC attesting to the destruction, inactivation, or transfer of PV materials. Facilities reporting materials that were not yet subject to containment (e.g., OPV/Sabin 1) could retain their inventory without registering as a PEF. Facilities reporting storage of potentially infectious materials subject to GAPIII/IV (e.g., WPV2/VDPV2 PIM) were not designated as PEFs by US NAC and have been excluded from this report. These facilities were requested to contact US NAC for additional guidance prior to starting work with these specimens.

### 2.3. Certification Process

**Figure 1:**
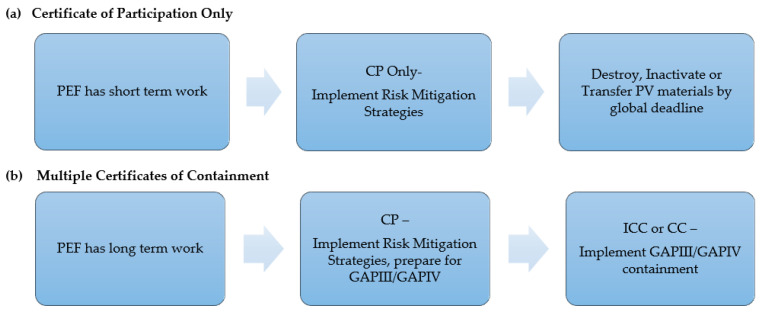
Two certification paths for the phased transition to WHO GAPIII/GAPIV containment in the United States: (**a**) certification path for PEFs planning to conclude work prior to the global CP expiry date; (**b**) certification path for PEFs planning to continue work and implement GAPIII/GAPIV containment. CP—Certificate of Participation; ICC—Interim Certificate of Containment; CC—Certificate of Containment.

As the first step in the certification process, GCC requested that facilities complete a Certificate of Participation (CP) application, provide a description of preliminary containment conditions/risk mitigation strategies used to safeguard PV materials, and provide a time-bound action plan describing progress in the implementation of GAPIII containment or a commitment to conclude work prior to the established global CP expiry date [14]. Once a facility’s CP application information was endorsed by US NAC, the application was submitted to WHO for additional processing and release to the GCC-CWG for review and GCC endorsement. Facilities that perform critical national or international activities with PV materials and plan to retain PV for an extended period were enrolled to be audited against GAPIII/GAPIV to become fully certified PEFs. Two paths for CP in the GAPIII/GAPIV PEF auditing scheme are available for a phased transition to WHO GAPIII/GAPIV containment in the United States (Figure 1).

### 2.4. Site Visits

Site visits were conducted for verification of facility CP application information and for periodic monitoring once the CP was awarded. Site visits consisted of laboratory tours, personnel interviews, completion of a facility questionnaire, and document review by US NAC auditors and CDC technical subject matter experts to assess preliminary containment conditions/risk mitigation strategies, laboratory design/features based on *Biosafety in Microbiological and Biomedical Laboratories* (BMBL) 5th edition guidance [21] and GAPIII [10], and inventory of PV materials during 2018–2022. In lieu of an onsite visit, two virtual site visits were conducted in October and November 2020 with adapted assessment criteria due to the COVID-19 pandemic. Findings were communicated to the facility in writing following each site visit, and, when applicable, the facility submitted information on corrective actions taken to resolve identified findings.

### 2.5. Information Collection and Analysis

CDC determined that the national survey, facility questionnaire, and CP application information collection activities conducted under the project are exempt from the requirements of the Paperwork Reduction Act (PRA) as they fall under the activities authorized under the National Childhood Vaccine Injury Act (NCVIA) in Section 2102(a)(6)-(a)(7) of the Public Health Service Act (42 USC 300aa-2(a)(6)-(a)(7). This activity was reviewed by CDC and was conducted consistent with applicable federal law and CDC policy. §See, e.g., 45 C.F.R. part 46, 21 C.F.R. part 56; 42 U.S.C. §241(d); 5 U.S.C. §552a; 44 U.S.C. §3501 et seq. Descriptive statistical analyses (median, frequency) on PEF data sets were performed in Microsoft 365 Excel v2102; maps were created in Microsoft Power BI v2.95.804.0.

## 3. Results

### 3.1. Facility Identification and Outreach

**Figure 2:**
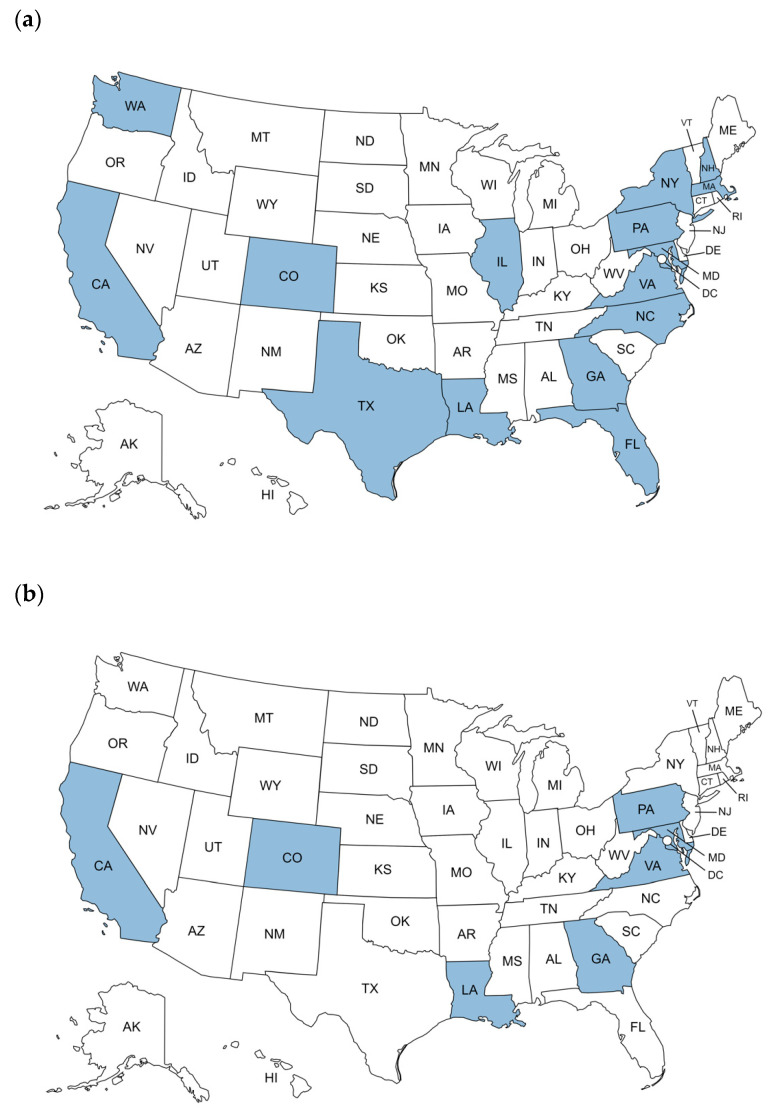
US states hosting poliovirus-essential facilities between 2017 and 2022: (**a**) US states hosting potential PEFs between 2017 and 2022 as reported by respondents to national poliovirus containment surveys or direct communication of facilities with CDC, and (**b**) US states hosting PEFs (WPV2/VDPV2, OPV2, and WPV3/VDPV3 materials) endorsed by US NAC and GCC as of 31 December 2022.

**Figure 3:**
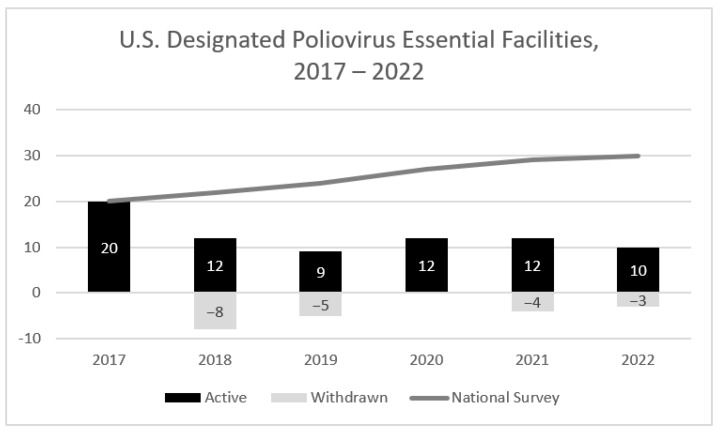
Trends in poliovirus-essential facilities designated in the United States, 2017–2022. Number of active and withdrawn PEFs by year compared to cumulative count of potential PEFs (N = 30) identified in national survey.

A total of 30 potential PEFs (retaining WPV2/VDPV2, OPV2, and/or WPV3/VDPV3 infectious materials) located in 15 states were identified via national poliovirus containment surveys and correspondence to CDC between 2017 and 2022. Following US NAC initial outreach, facility leadership at ten facilities (10/30, 33%) opted to destroy, inactivate, or transfer PV infectious materials rather than seek certification as a PEF. As of 31 December 2022, a total of 10 PEFs in seven states remained active in the containment certification process (Figure 2). The annual number of potential PEFs in the United States fluctuated between 2017 and 2022, with the largest decrease (20 to 12, 40% reduction) occurring in 2018 following initial US NAC outreach (Figure 3).

### 3.2. Certificate of Participation Application

US NAC developed a fillable CP application adapted from the WHO CP form [14]. The US NAC CP application collected facility information on essential work, virus type (wild, vaccine-derived, OPV, and novel oral polio vaccine (nOPV)), viral propagation volume, the number of essential personnel, laboratory locations, a time-bound action plan to either complete work under a CP or implement GAPIII/GAPIV, and final certification goal (i.e., plans to complete PV work or to be audited against GAPIII/GAPIV standards for certification as a PEF). The US NAC CP application required approval and signatures from the facility’s principal investigator, institutional biosafety officer, and a senior institutional representative (e.g., Director, University President) to affirm full commitment by facility partners to engage in the containment certification process.

A total of 20 potential PEFs submitted CP applications to US NAC as of 31 December 2022, where two of the facilities operated PV laboratories at different physical addresses. Two of twenty CP applications were withdrawn at the facilities’ request prior to US NAC verification of risk mitigation strategies and excluded from this analysis. Eighteen potential PEFs comprised academic, commercial, and government facility types. No PV vaccine manufacturers operate in the United States. Most US facilities (12/18, 67%) will complete critical work with PV prior to the CP global expiry date, while six facilities will seek additional GAPIII certification as PEFs.

### 3.3. Preliminary Containment Conditions—Risk Mitigation Strategies

An external US working group was established under a CDC federal advisory committee with subject matter expertise in biosafety, containment laboratories, public health, emergency response, and infectious diseases. Seven experts provided input to US NAC on best practices to safeguard polioviruses in domestic laboratories. In 2018, US NAC developed 35 laboratory risk mitigation strategies adapted from GAPIII elements and BMBL 5th edition guidance 21], and, in 2020, the list of risk mitigation strategies was updated with 15 additional containment strategies (Table 1). These strategies were developed in consultation with the external working group and feedback from PEFs. Risk mitigation strategies were selected based on gaps identified during US NAC facility outreach, focused on enhanced biosafety practices considered most likely to mitigate the risk of potential PV occupational exposure or release, and, in the 2020 revision, to address potential biosecurity and emergency management risks and expand containment to WPV3/VDPV3 infectious materials. US NAC provided training on risk mitigation strategies to facilities and requested progress on their implementation during the CP application process, at a minimum of two months later. The risk mitigation strategies were the recommended minimum safeguards for PV infectious materials in US laboratories effective until the global CP expiry date. An overview of the implementation timeline and US approach to enroll facilities in containment certification is shown in Figure S1.

### 3.4. Certification Process

The results for facilities identified in the national survey and participation in containment certification are shown in Table 2. The first step in the PEF containment certification process has multiple steps including (1) US NAC endorsement, (2) WHO review, (3) GCC-CWG review and recommendation to GCC, (4) GCC endorsement, and (5) US NAC issuance of CP to PEF. Prior to endorsement, US NAC assessed PEFs based on critical national or international work activities, laboratory features, and the implementation of preliminary containment conditions/risk mitigation strategies during site visits. US NAC also communicated with facilities to resolve incomplete CP application information, site visit findings, and requests for clarification resulting from reviews by the WHO and the GCC-CWG during the certification process.

Characteristics for eighteen PEFs (n = 20 PV laboratory sites), laboratory designs, and 27 laboratory features are shown in Table 3. US PEFs that enrolled in the CP process used and/or stored PV in A/BSL-2 (65%), A/BSL-3 (20%), and storage (15%) areas. Some PV laboratories had enhanced features, including GAPIII facility requirements such as a ventilation system maintaining inward directional airflow (55%), a double-door personnel airlock/anteroom (35%), a personal walk-through exit shower (10%), and an effluent decontamination system (5%) (Table 3).

### 3.5. Site Visits

During 2018–2022, US NAC conducted a total of 27 site visits at 20 PV laboratories in 18 PEFs to verify preliminary containment conditions/risk mitigation strategies. From June 2018 to June 2020, US NAC assessed 35 risk mitigation strategies and found US PEFs adopted preliminary containment conditions (median = 22.5; max = 30; min = 3) during 14 site visits. US NAC also found a similar trend (median = 29; max = 43; min = 15) when the number of risk mitigation strategies was increased to 50 (n = 13 visits), with an increase from a minimum of 3 to 15 strategies observed during these visits. The results for the risk mitigation strategies assessed and the PEF containment practices verified during site visits are shown in Table 1.

US NAC found PEFs separated the PV laboratory room from public areas with two doors (25/27, 93%), reduced the quantities of polioviruses stored (20/27, 74%), segregated PV from all other materials (22/27, 81%), and dedicated reagents used for PV work (15/27, 56%). In the United States, a limited number of PEFs perform animal studies with PV serotypes subject to containment, and, thus, animal containment criteria were not applicable to most facilities. US NAC observed animal-specific risk assessments and HEPA-filtered exhaust on containment caging systems at two PEFs performing animal work, but one of the two PEFs did not fully implement the recommended animal PV containment practices (i.e., a dedicated caging system, animal recordkeeping through final disposition, and emergency response plans for an escaped animal) (Table 1).

**Figure 4:**
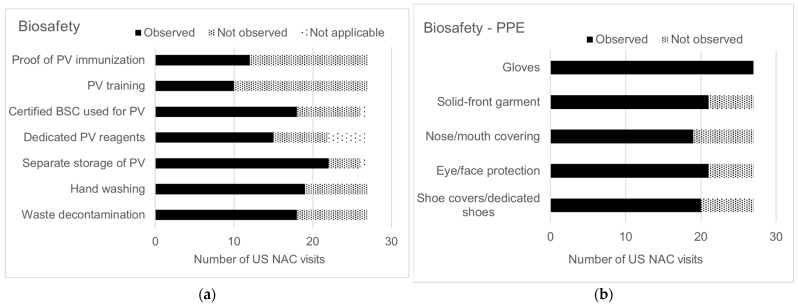

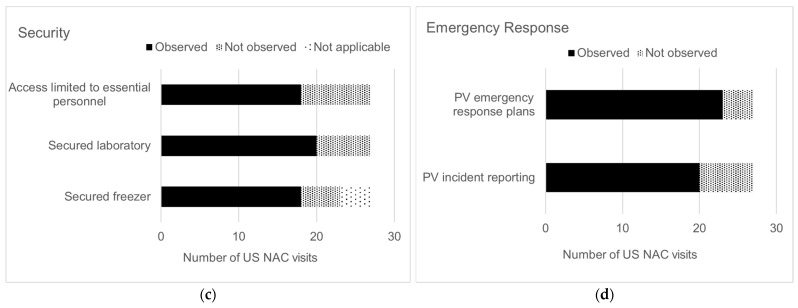
Risk mitigation strategies implemented by U.S. PEFs at time of site visit (n = 27), by category: (**a**) Biosafety category—seven key biosafety containment strategies. For storage-only PEFs or PEFs that did not store PV, certain containment strategies were not applicable. (**b**) Biosafety category—five personal protective equipment (PPE) containment strategies. (**c**) Security category—three containment strategies. For freezers located in a PV-dedicated secured laboratory, a secured freezer is not applicable. (**d**) Emergency response category—two containment strategies.

Areas for improvement included the use of certified primary containment devices for manipulation of PV materials, appropriate decontamination of solid and liquid biohazardous waste, hand hygiene practices, training, and proof of PV immunization records for essential personnel granted access to PV laboratories and materials (Figure 4). Some PV laboratory sites (8/20, 40%) fully implemented the recommended personal protective equipment (PPE) for laboratory staff. For PPE, a nose and mouth covering had the lowest implementation observed by US NAC during site visits (8/27, 30%) (Figure 4). Similarly, facility security controls and emergency response protocols needed modifications to address poliovirus containment (Figure 4). For example, US NAC observed that PV laboratories did not restrict access only to essential personnel for PV laboratory and storage areas (9/27, 33%). Facilities implemented corrective actions to resolve US NAC findings prior to issuance of CPs. US NAC also conducted periodic monitoring after the CP was awarded for several PEFs (7/18, 39%) to ensure containment practices were maintained.

### 3.6. US NAC and GCC Endorsement

National and global endorsement and associated processing times for facility CP applications are shown in Table 2. US NAC received completed CP application information (i.e., CP application, preliminary containment conditions/risk mitigation strategies, time-bound action plan, material inventory, resolution of site visit findings) for 17 of 18 PEFs. GCC-CWG requested clarification on eight applications and returned three CP applications as ‘unsatisfactory’, requiring PEF reapplication. A total of sixteen PEFs were endorsed by GCC in the United States as of June 2023. Extended processing times (median days = 233; min = 91; max = 678) were observed for US PEFs to achieve NAC- and GCC-endorsed CP applications (Table 2).

### 3.7. Withdrawal of PEFs

Following GCC endorsement, six PEFs completed essential work with WPV2, WPV3, and OPV/Sabin 2, and/or received a temporary waiver from WHO to exclude research activities with nOPV2 from the Containment Certification Scheme resulting in ten active PEFs as of 31 December 2022 (Figure 3). To withdraw from containment certification, PEFs submitted a written statement signed by the principal investigator or institutional representative requesting to withdraw from containment certification, documentation with two witness signatures of the transfer, inactivation or destruction of WPV2/WPV3 and OPV2 materials, and a description of validated decontamination methods used to decommission laboratory areas. US NAC reviewed the documentation and notified WHO of the withdrawal. Overall, a substantial reduction (20/30, 67%) in the number of potential US PEFs has occurred since the appointment of US NAC.

## 4. Discussion

Poliovirus containment is a key goal of the poliovirus eradication strategy and a prerequisite for future certification of a poliovirus-free world [22]. As of November 2023, WHO reported a total of 22 countries with plans to retain PV materials in 69 PEFs, with 42 designated PEFs awarded GCC-endorsed CPs to become audited against GAPIII [6,23]. While some countries experienced delays in appointing an NAC [6,23], US NAC was established in January 2018 following a process initiated by CDC. The rapid implementation of containment activities in the US was likely due to existing partnerships between CDC and WHO, monthly coordination meetings between US NAC and WHO, the establishment of an external working group, and allocation of CDC resources and full-time staffing to US NAC. These activities supported the engagement of PV laboratories in the certification process and safeguarded PV infectious materials with enhanced safety and security practices. Notably, all US PV laboratories reporting WPV2/VDPV2, OPV2, and WPV3/VDPV3 infectious materials in the national survey participated in the containment certification process or opted to destroy, inactivate, or transfer these materials to comply with GAPIII/GAPIV. We report on progress made towards a successful transition to more stringent GAPIII/GAPIV containment in the United States.

Poliovirus does not currently meet the criteria for classification as a select agent in the United States, and, as a result, the United States does not have a legal framework to require containment of polioviruses as of November 2023 [24]. Further, poliovirus has previously been classified as a risk group 2 agent with recommended biosafety level 2 (BSL-2) laboratories and containment practices as described in BMBL guidelines [21], whereas poliovirus containment is closely aligned with enhanced BSL-3 laboratories and practices [10,11]. These issues presented a challenge to the immediate implementation of GAPIII containment by US facilities performing essential work with polioviruses. Most US PEFs planned to complete work under a CP and not seek additional certification. Thus, the United States developed a phased approach to manage a voluntary program of facilities retaining WPV2/VDPV2, OPV2, and WPV3/VDPV3 to work with and store these viruses safely and securely while progressing toward stringent containment measures outlined in GAPIII/GAPIV.

The total number of PEFs in the United States fluctuated between 2017 and 2022, with the largest decrease occurring in 2018. The PEFs reported GAPIII containment requirements as a deterrent for the retention of PV materials. However, since 2019, a modest reduction in PEFs was balanced with newly identified facilities. Globally, the United States continues to have the largest number of facilities retaining polioviruses that entered WHO’s Containment Certification Scheme as of November 2023 [23]. Several US PEFs did not modify operations to comply with GAPIII containment, consistent with the 2018 WHA resolution [12], until contacted by US NAC. As a result, US NAC developed recommendations for preliminary containment conditions/risk mitigation strategies in consultation with an independent US working group and PEF partners that were realistic for facilities to achieve in a short time. The US NAC risk mitigation strategies were standardized for transparency and consistency in the certification process due to the large number of US PEFs. US NAC found that site visits and discussions with facility personnel were critical to raise awareness of containment requirements and to verify the implementation of practices prior to endorsement of CP applications.

US NAC also observed gaps in PEF biocontainment practices during site visits, including some fundamental GAPIII principles. For example, many PEFs had not verified PV immunizations for essential personnel, ensured stringent adherence to hand hygiene practices, or required use of certified primary containment devices for all manipulations of PV infectious materials. These site visit findings, along with a reported 21 incidents of PV release at research laboratories and vaccine production facilities that have occurred globally since 2000, resulting in 16 poliovirus infections [25], suggest current containment practices in PV laboratories should be reviewed and strengthened. US NAC site visits were instrumental in observing PV biocontainment practices, and although gaps were identified, facilities resolved findings and improved poliovirus containment practices in designated US PEFs. These results show that a transition from decades of poliovirus research using BSL-2 laboratories and practices to higher biocontainment standards required time, engagement, and collaboration by all partners, particularly for enhanced laboratory containment of attenuated OPV2 vaccine viruses [7].

US NAC found that its PEF certification process resulted in consistent implementation of enhanced safety and security practices beyond BSL-2 standards for PV infectious materials. US NAC acknowledges that the risk mitigation strategies (preliminary containment conditions) reported here were not a substitute for WHO’s GAPIII/GAPIV containment requirements. In addition, the approach taken was risk-based and may not be applicable to all PEF-hosting countries globally. For example, poliovirus vaccines are not manufactured in the United States; this is a higher risk activity with multiple reported containment breaches [9,25,26,27]. The United States also complies with GAPIII/GAPIV requirements for national high population immunization coverage (93% inactivated poliovirus vaccine, third dose, as of 2022 [28,29]) as well as sanitation system safeguards to mitigate the transmission of diseases such as those due to wild polioviruses.

US NAC continues to improve its guidance for PEFs and the risk mitigation strategies were updated with more descriptive information in 2020–2021, including containment of WPV3/VDPV3 and additional emergency response and security strategies aligned to the GAPIII/GAPIV standard. US NAC identified similar gaps in PEF readiness for these enhancements. As a result, PEF readiness for site visits and the implementation of recommended enhanced containment conditions were factors that contributed to delays in the certification process. While our recommended preliminary containment conditions were adapted from GAPIII, PEF conformance to a more complex and system-based GAPIII/GAPIV standard suggests that additional time may be necessary for PEFs to achieve full conformance to GAPIII/GAPIV than currently outlined in the WHO Containment Certification Scheme [14]. Most PEF-hosting-country NACs have been established; however, no designated PEFs have been fully certified to GAPIII/GAPIV containment worldwide [6,23].

Poliovirus-essential facility CP applications required nearly 8 months (median days = 233; range 91–678) for endorsement by both US NAC and GCC from the date of submission to US NAC. Currently, the WHO Containment Certification Scheme recommends that NACs review CP applications to determine if the PEF can potentially meet GAPIII criteria, but does not specify site visit assessments for this first step [14]. US NAC incorporated site visits prior to its endorsement of PEFs, a significant contributing factor in longer processing times. While PEFs averaged less than three months to implement enhanced containment practices, one PEF required 418 days to implement the recommended risk mitigation strategies due to limited availability of BSL-3 space during the COVID-19 pandemic. Despite a longer processing time, US NAC deemed these visits necessary before giving its endorsement of a designated PEF and instrumental in responding to WHO and/or GCC requests for clarification during their review processes. US NAC also found longer processing times were needed when GCC returned a few CP applications as unsatisfactory and requiring reapplication. These CP applications required additional correspondence with WHO and GCC for resolution of reviewer feedback and resulted in an extended approval process. The CP processing times improved to less than six months for four US PEFs enrolled during 2020–2022, suggesting that, despite challenges and the COVID-19 pandemic, US NAC’s processes are effective for the identification and certification of PEFs.

Overall, the United States successfully appointed an NAC, reduced the number of potential PEFs by 67%, and ensured all facilities retaining WPV2/VDPV2, OPV2, and WPV3/VDPV3 infectious materials in the national survey submitted CP applications. Notably, the United States is complying with the May 2018 World Health Assembly resolution 71.16 [12] without a national legal framework requiring containment of polioviruses. As of June 2023, one US PEF achieved an Interim Certificate of Containment against GAPIII to advance beyond the initial CP process ([30] unpublished data, manuscript in preparation). The United States is one of only three countries achieving this accomplishment. The results reported suggest that US NAC’s outreach and phased approach for the implementation of stringent GAPIII/GAPIV containment measures, engagement of laboratory partners in preliminary containment recommendations, and to raise facility awareness of the risks posed by retaining eradicated polioviruses has led to the adoption of enhanced laboratory safeguards. US NAC will continue to monitor its national survey, PEFs, and report on the transition to GAPIII/GAPIV containment audits aligned with the anticipated global CP expiry date.

### Limitations

The findings in this report are subject to at least three limitations. First, we were unable to account for possible under-reporting of facilities retaining poliovirus materials in national surveys used for the identification of PEFs. Second, and related to the first limitation, the United States does not maintain a registry of laboratories, and thus the unknown total number of laboratories limited the ability to perform an independent verification of laboratories retaining poliovirus materials. Third, the GAPIII and BMBL guidelines were sampled to develop risk mitigation strategies focused on biosafety practices, and thus do not represent other mitigation measures that PEFs may use to reduce risks in the retention of polioviruses.

## 5. Conclusions

The United States made noteworthy progress on the implementation of the WHO GAPIII/GAPIV Containment Certification Scheme. Globally, the United States was the second country to receive an endorsement of poliovirus-essential facility CP applications by the GCC and has the highest number of PV facilities endorsed as designated poliovirus-essential facilities as of June 2023. US PEFs established a community of practice committed to the implementation of containment certification and adopted enhanced biosafety and biosecurity containment practices for PV materials exceeding routine BSL-2 practices during the transition to WHO GAPIII/GAPIV containment, and US NAC engaged institutional leadership, biosafety professionals, and investigators in global poliovirus containment goals.

## Figures and Tables

**Table 1:** US National Authority for Containment’s risk mitigation strategies for poliovirus materials, 2018–2022.

No.	Hazard Control ^a^	Category	Risk Mitigation Strategy for Poliovirus Materials ^b^	Containment Strategies Observed by US NAC	
				Frequency/No. Visits	Percent(%)
1	Elimination	Biosafety	Destroy unneeded poliovirus materials	20/27	74
2	Elimination	Biosafety	Inactivation, fixation or extraction of poliovirus materials	8/27	30
3	Engineering	Biosafety	Dedicated room (e.g., an isolation room within a larger laboratory) used for poliovirus	8/27	30
4	Engineering	Biosafety	Two doors are present between public areas and the laboratory room	25/27	93
5	Engineering	Biosafety	Certified BSC used for poliovirus work	18/27	67
6	Engineering	Biosafety	Centrifuge safety cups/sealed rotors that are loaded and unloaded in a BSC for poliovirus work	11/27	41
7	Engineering	Biosafety	Containment caging system with HEPA filtered exhaust used for housing PV-infected animals	2/13	15
8	Administrative	Biosafety	Risk assessments for poliovirus containment	9/13	69
9	Administrative	Biosafety	Risk assessments for animal work	2/13	15
10	Administrative	Biosafety	PV work reviewed/approved by institutional committees (i.e., institutional biosafety committee (IBC), institutional animal care and use committee (IACUC))	4/13	31
11	Administrative	Biosafety	Dedicated BSC and incubator used for poliovirus	10/27	37
12	Administrative	Biosafety	Dedicated caging system used for PV-inoculated animals	1/13	8
13	Administrative	Biosafety	Shared laboratory uses PV spatial and temporal separation with appropriate decontamination procedures	9/27	33
14	Administrative	Biosafety	Personnel trained in poliovirus biosafety and security practices	10/27	37
15	Administrative	Biosafety	Personnel receive annual refresher training	5/13	38
16	Administrative	Biosafety	Personnel provide proof of poliovirus immunization	12/27	44
17	Administrative	Biosafety	Personnel enrolled in occupational health program	12/27	44
18	Administrative	Biosafety	Personnel competent in good microbiological techniques	17/27	63
19	Administrative	Biosafety	Personnel wash hands prior to exit of the laboratory	19/27	70
20	Administrative	Biosafety	Protective clothing and gloves removed prior to exit from the laboratory	16/27	59
21	Administrative	Biosafety	Reusable PPE is decontaminated prior to storage and reuse	12/27	44
22	Administrative	Biosafety	Disposable PPE is treated as biohazardous waste	22/27	81
23	Administrative	Biosafety	Durable leak proof transport container used when poliovirus is removed from primary containment	17/27	63
24	Administrative	Biosafety	Dedicated reagents used for poliovirus	15/27	55
25	Administrative	Biosafety	Segregate poliovirus from all other materials (e.g., in own clearly labeled freezer box)	22/27	81
26	Administrative	Biosafety	Perform work with one poliovirus serotype at a time to minimize potential cross-contamination (when possible)	6/13	46
27	Administrative	Biosafety	Decontamination of work surfaces	18/27	67
28	Administrative	Biosafety	All materials leaving the laboratory are decontaminated using an appropriate method (autoclave, incinerator)	18/27	67
29	Administrative	Biosafety	Periodic validation of autoclave decontamination procedures	21/27	78
30	Administrative	Biosafety	Containers with poliovirus are surface disinfected prior to removal from the BSC	13/27	48
31	Administrative	Biosafety	Procedures for decontamination of equipment are implemented	14/27	52
32	Administrative	Biosafety	Chemical treatment known to inactivate poliovirus implemented	7/27	26
33	Administrative	Security	Locked freezer where poliovirus is stored (when stored outside a dedicated, secured laboratory)	18/27	67
34	Administrative	Security	Locked laboratory	20/27	74
35	Administrative	Security	Limit access to personnel identified as essential by the facility	18/27	67
36	Administrative	Security	Poliovirus inventory records are current, accurate and complete	7/13	54
37	Administrative	Security	Poliovirus animal tracking and infected tissue inventory records	1/13	8
38	Administrative	Security	Security policies implemented for controlled access to poliovirus materials and areas	9/13	69
39	Administrative	Security	Individual entries into poliovirus areas are documented (e.g., electronic record, manual logbooks)	8/13	61
40	Administrative	Security	Visitor policy for entering poliovirus areas	6/13	46
41	Administrative	Emergency Response	Emergency response plans developed, including measures to protect personnel and the environment in the event of a release ^c^	23/27	85
42	Administrative	Emergency Response	Personnel report accidents or incidents with PV per institutional policy	20/27	74
43	Administrative	Emergency Response	Notify appropriate state and local agencies of possession of PV materials	8/13	61
44	Administrative	Emergency Response	Develop and coordinate emergency response plans with first responders	7/13	54
45	Administrative	Emergency Response	Develop response procedures for PV-infected escaped animals	1/13	8
46	PPE ^d^	Biosafety	Protective laboratory clothing with a solid-front (e.g., disposable wrap-around gown, scrubs, coverall)	21/27	78
47	PPE	Biosafety	Gloves (double gloves are recommended)	27/27	100
48	PPE	Biosafety	Face or surgical mask or respirator	19/27	70
49	PPE	Biosafety	Eye and face protection for anticipated splashes or sprays (e.g., safety glasses, face shield)	21/27	78
50	PPE	Biosafety	Shoe covers or dedicated shoes	20/27	74

^a^ Adapted from Hierarchy of Controls = elimination, substitution, engineering controls, administrative controls, and personal protective equipment (PPE) in Hierarchy of Controls Workplace Safety & Health Topics website; CDC/The National Institute for Occupational Safety and Health (NIOSH), 2015. Available at https://www.cdc.gov/niosh/topics/hierarchy/default.html (accessed on 6 December 2023). ^b^ U.S. NAC risk mitigation strategies (*n* = 35) were implemented for poliovirus type 2 in 2018; additional risk mitigation strategies (*n* = 15, numbers 7–10, 12, 15, 26, 36–40, 43–45) were implemented in late 2020 for WPV2/VDPV2, OPV2, and WPV3/VDPV3 materials. ^c^ In October 2021, the risk mitigation strategy for emergency response plans was updated to align response procedures for potential poliovirus exposures with WHO guidance. ^d^ PPE—personal protective equipment.

**Table 2:** US poliovirus-essential facilities’ participation in containment certification and application processing times, 2018–2022.

CCS ^a^ Process	PEF Participation in Certification	Frequency (N = 30)	Percent (%)	Time (Days)	
				Median	Range
Option A	Facility submits CP application	20	67	-	-
USA requirement	i. Implement risk mitigation strategies	18/20	90	83	3–418
Step 1	ii. NAC endorsement	17/20	85	84	3–418
Step 2	iii. WHO review	17/20	85	10	1–128
Step 3	iv. GCC-CWG review	17/20	85	22	1–77
Step 4	v. GCC endorsement ^b^	16/20	80	12.5	1–463
Step 5	vi. CP issued ^c^	15/20	75	233	91–678
Step 6	vii. Facility withdraws from certification ^d^				
-	• Before US NAC endorsement	3/20	15	39	38–373
-	• Before GCC endorsement	1/20	5	608	-
-	• Ends work under valid CP	6/20	30	841.5	151–1471
Option B	No participation, destroy or transfer PV	10	33	-	-

^a^ CCS—Containment Certification Scheme [14]. ^b^ GCC returned 3 applications as ‘unsatisfactory’; 2 of 3 facilities reapplied and received GCC endorsement. ^c^ One GCC-endorsed PEF plans to destroy WPV2 and WPV3 and retain WPV1 only, US NAC issuance of certificate pending (data as of June 2023). ^d^ Three potential PEFs withdrew from containment certification scheme prior to US NAC endorsement (n = 2 destroyed PV materials, n = 1 opted not to receive PV materials). One PEF opted to transfer PV materials to another PEF instead of reapplication to achieve GCC endorsement. Six PEFs concluded work with containment PV strains under a valid CP and were withdrawn from containment certification as of 31 December 2022.

**Table 3:** Characteristics, laboratory design, and laboratory features of US poliovirus-essential facilities (PEFs).

PEF Characteristics ^a^			27 Laboratory Features ^b^		
N = 18 PEFs	Frequency (N = 18)	Percent (%)	PV Laboratory Sites (Operated in 18 PEFs)	Frequency (n = 20)	Percent (%)
*Facility primary work objective*			*Containment boundary*		
Biomedical research	9	50	Containment perimeter sealable for gaseous decontamination	5	25
Clinical diagnostic laboratory	1	6	Facility is equipped with a double-door personnel airlock/anteroom	7	35
Public health laboratory	4	22	Double doors are interlocked (physical or procedural)	5	25
Industrial/production laboratory	2	11	Backflow prevention on all services/utilities passing across the boundary	2	10
Other	2	11	*Sinks and Showers*		
*Virus type ^c^*			Hands-free/automated hand washing sink	8	40
WPV2/VDPV2	9	50	Personal exit shower	4	20
WPV3/VDPV3	6	33	Personal walk-through exit shower	2	10
OPV/Sabin 2	12	67	Emergency shower	16	80
nOPV2	6	33	*Ventilation System*		
*Work type(s) ^d^*			Controlled air system maintains inward directional airflow	11	55
Research	12	67	Exhaust air is HEPA filtered	7	35
Vaccine production	0	0	Dedicated ventilation system to PV area (exhaust and supply)	2	10
Clinical trials	3	17	Backflow protection on supply air	4	20
Animal model	4	22	Ductwork sealable for gaseous decontamination	4	20
Diagnostics	3	17	Monitors/alarms to ensure directional airflow can be readily validated	5	25
QC testing ^e^	3	17	*Decontamination Systems*		
Storage only	3	17	Single door autoclave	7	35
*Certification goal ^f^*			Pass-through autoclave	5	25
Certificate of Participation (CP)	12	67	Material airlock/decontamination chamber sealable for gaseous decontamination	1	5
Interim Certificate of Containment (ICC)	1	5	Dunk tank containing sufficient active compound to inactivate poliovirus	1	5
Certificate of Containment (CC)	5	28	Effluent decontamination system	1	5
			*Security*		
			Entry door(s) equipped with lock/lock cylinder rated as burglary resistant	17	85
*Laboratory Design*	Frequency (n = 20)	Percent (%)	Lock(s) fail secure and allow egress only	11	55
Design ^g^			Locked door with two-factor access control measure	6	30
A/BSL-3	4	20	Video surveillance	13	65
A/BSL-2	13	65	Two-person system for PV work	7	35
Storage only	3	15	Intrusion detection system	8	40
			Facility perimeter is subject to constant monitoring	13	65
			Facility is located on a secure site with perimeter control	9	45

^a^ PEFs (N = 18, operated in 20 PV laboratory sites) with CP applications and preliminary containment conditions verified by US NAC. ^b^ Physical laboratory features, when available, were reported with a list of preliminary containment conditions to GCC. These 27 laboratory features were not required by US NAC for endorsement of a CP. ^c^ WPV—wild poliovirus; VDPV—vaccine derived poliovirus; OPV—oral polio vaccine; nOPV—novel oral polio vaccine. Ten PEFs retain a combination of virus types. ^d^ Work type(s)—11 PEFs with one work type, 7 PEFs with more than one work type. ^e^ QC—quality control. ^f^ Certification goal—three facilities changed between CC- and CP-only goals, one facility changed from ICC- to CP-only goal compared to initial CP applications received. ^g^ A/BSL = animal/biosafety level. Data as of 31 December 2022.

## Data Availability

The datasets generated and analyzed during the current study are not publicly available due to privacy concerns for the poliovirus-essential facilities. Aggregated data are available from the corresponding author on reasonable request.

## Supplementary Materials

The following supporting information can be downloaded at https://www.mdpi.com/article/10.3390/pathogens13020116/s1: Figure S1: Poliovirus containment timeline and steps for enrollment of facilities in containment certification, United States.
