# Supplementary material for: Establishment of a Poliovirus Containment Program and Containment Certification Process for Poliovirus-Essential Facilities, United States 2017–2022
Source: Pathogens. 2024 Jan 27;13(2):116. doi: 10.3390/pathogens13020116 (PMC10893385; doi:10.3390/pathogens13020116)
Supplement: Supplementary file 1 [file pathogens-13-00116-s001.zip › pathogens-2825390-supplementary.pdf]

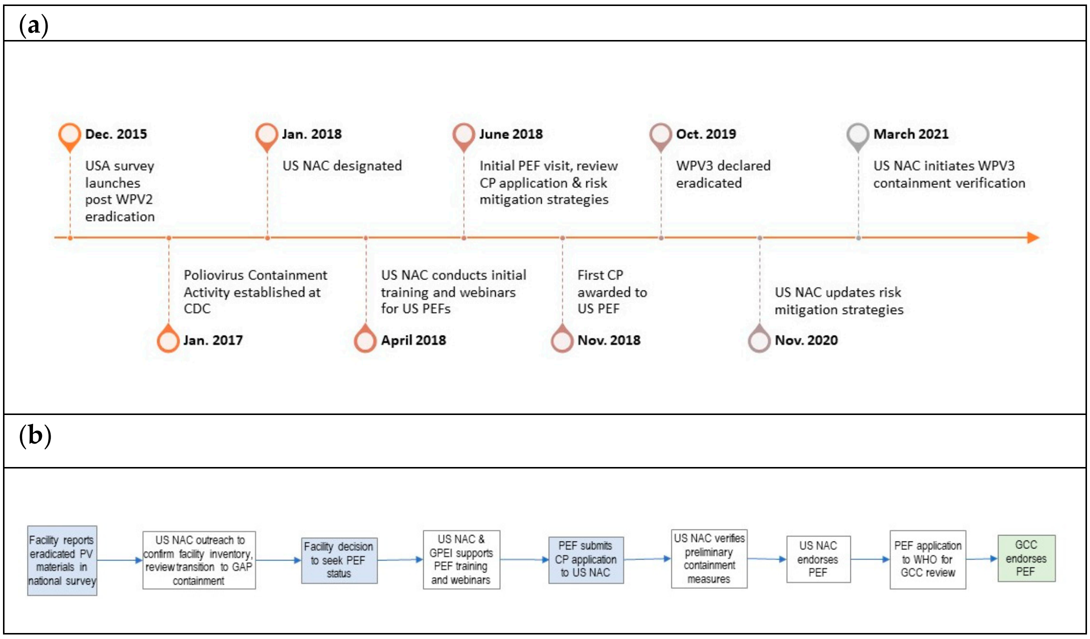

Figure S1. Poliovirus containment timeline and steps for enrollment of facilities in containment certification, United States. (a) Timeline of poliovirus containment implementation, such as establishment of the NAC and first CP awarded, for eradicated polioviruses in the United States; (b) Summary of US NAC process for enrollment of facilities in containment certification. Facility (blue) and US NAC (white) steps from identification to PEF endorsement are shown. US NAC and/or the Global Polio Eradication Initiative (GPEI) supported PEF training and webinars.
